# Niraparib Demonstrates Therapeutic Potential in Multiple Sclerosis through Inhibition of IL-17A Receptor Interaction and Promotion of Remyelination

**DOI:** 10.1021/acschemneuro.5c00519

**Published:** 2025-09-09

**Authors:** Muge Didem Orhan, Lalehan Oktay, Ayşe Irem Cınar, Aybek Kagan Yesil, Huseyin Tunc, Fatih Eren, Serdar Durdagi, Timucin Avsar

**Affiliations:** † Neuroscience, Graduate School of Education, Bahçeşehir University, Istanbul 34353, Turkey; ‡ Department of Medical Biology, Faculty of Medicine, 472597 Bahçeşehir University, Istanbul 34353, Turkey; § Neurooncology Laboratory, Faculty of Medicine, Bahçeşehir University, Istanbul 34353, Turkey; ∥ Computational Biology and Molecular Simulations Laboratory, Department of Biophysics, Faculty of Medicine, 52946 Bahçeşehir University, Istanbul 34353, Turkey; ⊥ Lab for Innovative Drugs (Lab4IND), Computational Drug Design Center (HITMER), Bahçeşehir University, Istanbul 34353, Turkey; # Neuroscience Program, University of Bonn, Bonn 53113, Germany; ∇ Department of Biostatistics and Medical Informatics, 52946 Bahçeşehir University, Istanbul 34353, Turkey; ○ Department of Medical Biology, School of Medicine, 64050 Marmara University, Istanbul 34353, Turkey; ◆ Faculty of Medicine, Recep Tayyip Erdoğan University, Rize 53020, Turkey; ¶ Institute of Gastroenterology Liver Research Unit, Marmara University, Istanbul 34353, Turkey; ◮ Molecular Therapy Lab, Department of Pharmaceutical Chemistry, School of Pharmacy, Bahçeşehir University, Istanbul 34353, Turkey

**Keywords:** multiple sclerosis, IL-17A, niraparib, PARP-1 inhibitor, cuprizone mouse model, demyelination

## Abstract

IL-17A is a pro-inflammatory cytokine that significantly contributes to the pathogenesis of autoimmune diseases, including multiple sclerosis (MS). Previous studies have suggested that PARP-1 inhibitors can modulate IL-17A-mediated inflammation, prompting the investigation of Niraparib, an FDA-approved PARP-1 inhibitor, as a potential therapeutic agent for MS. In this study, we hypothesized that Niraparib could disrupt the interaction between IL-17A and its receptor, IL-17RA. To evaluate this, we employed a binary quantitative structure−activity relationship (QSAR) model against anti-inflammatory diseases, which indicated Niraparib’s potential efficacy against MS. In silico analyses were conducted to identify key interaction sites and critical amino acid residues involved in the IL-17A/IL-17RA binding. Molecular docking simulations demonstrated Niraparib’s capability to interfere with these interactions. It has demonstrated significant efficacy in inhibiting the interaction between the IL-17A ligand and its receptor via reporter assay. In vivo assessments were performed using a cuprizone-induced demyelination model. Immune profiling revealed modulation of various T cell subsets and B cells, while cytokine analysis indicated a shift in inflammatory responses. Histological evaluations confirmed reduced demyelination and enhanced remyelination in affected brain regions. These findings support Niraparib’s potential as a therapeutic option for MS, warranting further exploration of its mechanisms and clinical relevance.

## Graphical abstract

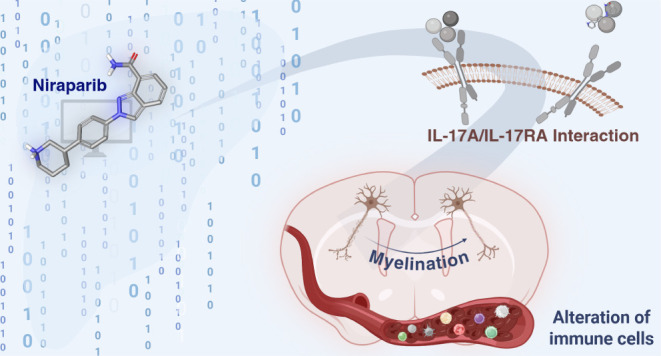

## Introduction

Multiple sclerosis (MS) is a chronic inflammatory, demyelinating, and neurodegenerative disorder of the central nervous system (CNS). It is characterized by the destruction of myelinated axons and the loss of myelin sheaths, which triggers an inflammatory response that damages oligodendrocytes (OLGs) and axons. This damage can result in axonal loss, and neurological impairment that range from temporary dysfunction to permanent deficits.^1^ MS typically occurs as localized inflammatory lesions in white matter and can be classified into different phenotypes as relapsing-remitting, primary, and secondary progressive MS.^2,3^ The exact pathogenesis of MS remains unclear, but it is recognized as an autoimmune disorder driven by autoreactive CD4+ T cells targeting myelin antigens. These T cells initiate an inflammatory cascade that leads to demyelination and axonal loss, with cytokines and inflammatory mediators.^1^ Various immune cells and their cytokines like IFN-γ, IL-17, IL-22, IL-1, IL-12, and TNF-α ^4–6^ are involved in MS lesions. IL-17A and Th17-related proteins are highly expressed in MS plaques and are the most transcribed cytokines in MS lesions. IL-17A promotes the production of additional proinflammatory cytokines and chemokines, enhancing strong proinflammatory effects.^1,7^ Th17 cells infiltrate the CNS by crossing the blood-brain barrier (BBB) and producing IFN-γ. This infiltration exacerbates tissue inflammation and leads to further immune cell accumulation and tissue damage.

IL-17A plays an important role in MS pathogenesis. Th-17 and other cells in the CNS produce IL-17A, and this cytokine activates different signaling pathways when it interacts with its receptor.^8^ The binding of IL-17A to its receptor activates microglia and astrocytes, allowing the development and progression of the disease. For this reason, the IL-17 pathway is now an important drug target in many autoimmune and chronic inflammatory diseases, and monoclonal antibodies targeting IL-17A, IL-23, IL-1β and related receptors are effective drug groups used in the treatment of the disease.^8–11^ Secukinumab is the IL-17A neutralizing monoclonal antibody and phase II trials demonstrated reduced MS lesions by blocking IL-17A.^12^ Additionally, rituximab, which is an anti-CD20 antibody, reduced IL-17A production and other cytokines in the EAE model, leading to decreased disease progression.^13^ Neutralizing IL-17A reduced pathogenic cytokine induction and significantly limited EAE progression.^14^ Innovative strategies that can effectively inhibit IL-17A may have great potential to promote remyelination and protect against neurodegeneration. Research into the inhibition of IL-17A has expanded to include various small molecules and peptides that target the IL-17A receptor, demonstrating potential efficacy in modulating inflammatory responses associated with autoimmune diseases, thus offering alternative therapeutic strategies beyond conventional monoclonal antibody treatments. These studies generally aim to discover effective small molecules, peptides and macrocycles targeting IL-17A/IL-17 RA protein−protein interaction (PPI). Currently, two patented small molecules are under investigation in clinical studies aimed at evaluating their efficacy in inhibiting IL-17A.^15^ However, these molecules have not been approved by the FDA yet. While the experimental autoimmune encephalomyelitis (EAE) model more accurately recapitulates the immune-mediated mechanisms of MS,^16,17^ this study primarily aimed to assess the effects of Niraparib on IL-17A inhibition, independent of peripheral immune cell infiltration. The cuprizone (CPZ) model, a well-established and reproducible model of toxin-induced demyelination^18,19^ was selected to allow a focused investigation of central nervous system (CNS)-intrinsic mechanisms, such as oligodendrocyte viability^20^ and myelin repair,^21^ without the confounding effects of systemic immune activation.^22^ Furthermore, elevated level of IL-17A cytokine in CPZ treated mice was shown.^23^ Therefore, although the EAE model better reflects the autoimmune pathology of MS,^16,17^ the primary objective here was to investigate the in vivo potential of Niraparib on demyelination using the CPZ model, which facilitates the evaluation of the drug’s direct effects on myelin and remyelination.^21,24^ This approach enabled us to better understand the potential protective or therapeutic effects of Niraparib in the context of toxic injury-induced demyelination.^25^

In addition to ongoing efforts in novel drug discovery, the repurposing of FDA-approved drugs as inhibitors of IL-17A has emerged as a critical area of contemporary research, underscoring their potential utility in the treatment of autoimmune disorders. Among these, poly­(ADP-ribose) polymerase-1 (PARP-1) inhibitors have gained considerable interest in drug repurposing studies due to their multifunctional properties, which suggest promising applications across various therapeutic contexts. Currently, PARP-1 inhibitors are widely recognized as targeted chemotherapeutic agents for breast and ovarian cancer. Specifically, in the context of BRCA-mutated breast cancer, these inhibitors exhibit cytotoxic effects on cancer cells by impeding PARP-1, an enzyme integral to DNA damage repair.^26,27^ To date, four PARP-1 inhibitors—Olaparib, Niraparib, Rucaparib, and Talazoparib—have received FDA approval for the treatment of pancreatic, breast, prostate, and ovarian cancers. Emerging evidence suggests that these agents also possess neuroprotective effects, prompting investigations into their potential role in inflammatory diseases, particularly those mediated by IL-17A, such as MS.^28,29^ In this regard, a binary quantitative structure−activity relationship (QSAR) model has been employed to predict the therapeutic potential of Niraparib, indicating its promising activity. Consequently, current study hypothesizes that Niraparib may function as an IL-17A/IL-17RA binding inhibitor, facilitating the treatment of demyelinating MS through a comprehensive approach that encompasses in silico molecular simulations alongside in vitro and in vivo studies.

## Results

### Molecular Docking Demonstrated That Niraparib Effectively Targeted the Protein−Protein Interaction between IL-17A and IL-17RA

To elucidate the interactions that inhibit IL-17A binding to IL-17RA via Niraparib, we docked the compound to the four regions previously identified by Liu et al. Residues involved in crucial interactions between IL-17A and IL-17RA were used to create docking grid boxes. The four binding sites identified by Liu et al., on the IL-17A/IL-17RA PPI interface encompass critical regions in both the N-terminal (Region I and Region II) and C-terminal (Region III and Region IV).^30^ Region I consists of S64 and R101, Region II consists of D42, R55, V65, and W67, Region III consists of Y85 and H86, and region IV consists of I127, V128, H129, H130, and V131 ^30^ (Figure 1a). Grid boxes were created using the centroid of these residues. These regions were considered key binding sites for potential protein−protein binding inhibitors, and further analyses were conducted with Niraparib to decipher the binding mechanism.

The anti-inflammatory potential of Niraparib was measured through binary QSAR-based therapeutic activity prediction using the Metacore/Metadrug platform. The anti-inflammatory therapeutic activity value (TAV) of Niraparib was calculated as 0.56. This presents an indicator for assessing the potential anti-inflammatory effects of Niraparib.

The docking scores of Niraparib at regions I, II, III, and IV were −4.38, −4.48, −5.30, and −6.58 kcal/mol, respectively. To further investigate the lowest energy docking pose of Niraparib at region IV, a 100 ns MD simulation was conducted. The interaction between IL-17A and Niraparib was monitored throughout the molecular dynamics (MD) simulation. These interactions were categorized by type, as shown in the figure: Hydrogen Bonds, hydrophobic, ionic, and water bridges. The bar graph has been normalized over the trajectory (Figure 1c); a value of 0.2 indicates that a specific interaction was sustained for 20% of the simulation duration. The timeline of interactions and contacts displayed in the bar graph is illustrated in Figure 1b. Each trajectory square indicates which residues interact with the ligand Niraparib. Some residues, such as P122, D123, L264, and E155 of IL-17RA and S40 on IL-17A chain A, have engaged in multiple specific contacts with Niraparib and have been maintained for most of the simulation (Figure 1 b,c). A 2D quantitative ligand interaction diagram indicates the interactions that were present for over 15% of the simulation time. Specifically, E155, W172, H266, D123, P122, and L264 were stabilizing Niraparib in the binding pocket. The MD simulation revealed interchanging interactions of Niraparib with the reported binding site residues on IL-17A, consisting of residues 127−131 on the A chain. Among these, hydrophobic interactions and water bridges with the backbone carbonyl of H129 were sustained throughout most of the simulation (Figure 1b,c).

Niraparib maintains strong hydrophobic contacts with L264 on IL-17RA throughout the simulation. A 2D quantitative ligand interaction diagram illustrates interactions that were present for over 15% of the simulation time. Specifically, E155, W172, H266, D123, P122, and L264 were responsible for stabilizing Niraparib in the binding pocket (Figure 1d). Most interactions with Niraparib occur through its carboxamide moiety, which stabilizes the molecule within the binding pocket via salt bridges and hydrogen bonds with water molecules. Notably, the rotatable bond between the benzene ring and piperidine moiety does not undergo dramatic conformational changes throughout the simulations, due to the surrounding interacting residues, specifically D123 and P122 on IL-17 and V128 on IL-17A (Figure 1d).

The root-mean-square deviation (RMSD) plot indicates protein backbone stability throughout the 100 ns MD simulation (Figure 1f). The LigFitProt RMSD of Niraparib, shown on the same plot, reflects the ligand’s translational RMSD relative to its initial pose in reference to the protein (Figure 1f).

**Figure 1:**
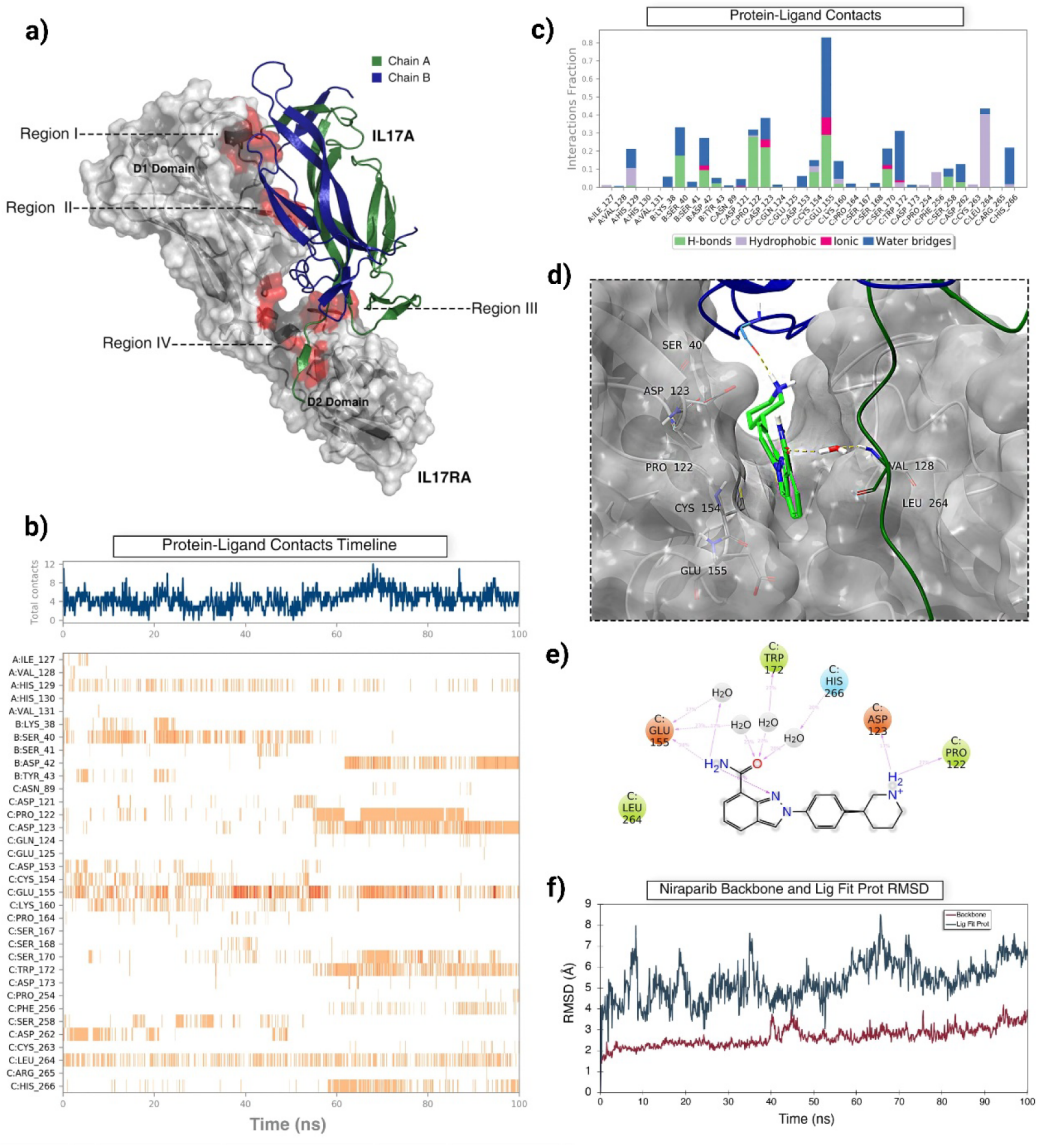
a) The IL-17A/IL-17RA complex (PDB ID: 4HSA)^30^ features chain A of IL-17A depicted in forest green and chain B in deep blue. IL-17RA is shown in a gray surface representation, with Regions I, II, III, and IV highlighted in red on the IL-17RA surface. b) A timeline representation illustrates the interactions and contacts between protein and ligand formed during the simulation. The top panel shows the total number of contacts made throughout the MD simulation. The bottom panel provides a detailed representation of the specific residues in Region IV that interact with Niraparib. c) The fraction of interactions and characterization of Region IV residues with Niraparib is presented. d) A close-up of the representative frame from the 100 ns MD simulation of Niraparib in Region IV is displayed, showcasing crucial residues and the binding mode of Niraparib. Chain A of IL-17A appears in forest green, while chain B is shown in deep blue alongside the gray surface representation of IL-17RA. e) A 2-D ligand interaction diagram depicts Niraparib in Region IV. This figure was obtained from the MD simulation, and the percentages represent the proportion of simulation time during which contacts with the residues were made. f) The protein backbone and LigFitProt RMSD of the MD simulation are also presented.

### Niraparib Significantly Blocked IL-17A and IL-17 Receptor Interactions in an In Vitro Assay, Showing Considerable Efficacy

The IL-17 reporter assay demonstrated the inhibition between IL-17A and its receptors (IL-17RA and IL-17RC). This assay allows for the measurement of the level of SEAP, which is released into the medium through the interaction of IL-17A, present in the medium, with IL-17R, which is overexpressed on the HEK cell surface. In the presence of IL-17A inhibitors, these inhibitors bind to IL-17A, preventing its binding with IL-17R and thus blocking SEAP release.100 μM Niraparib exhibited the maximum inhibitory effect at 99.3%. At 1 μM, the inhibitory efficacy was 22%. In comparison, 10 μM of the drug demonstrated 51% inhibitory activity (Figure 2a). Alongside the reporter assay, the IC_50_ value of Niraparib was calculated to be 70 μM, which is an acceptable concentration for cell culture experiments (Figures 2b and S1). A cell viability assay was also conducted to determine whether Niraparib has any toxic effects on cell survival at the desired concentrations. It was found that Niraparib posed no toxic effect after 24 h of treatment across all concentrations. However, treatment over 48 and 72 h resulted in significant cell death only at the 100 μM concentration (Figure 2c). Morphological assessments of the cells also indicated that there were no stress-induced morphological changes at concentrations lower than 100 μM (Figure S2). Overall, our data suggests that Niraparib inhibits ligand−receptor interactions without adversely affecting cell survival.

**Figure 2:**
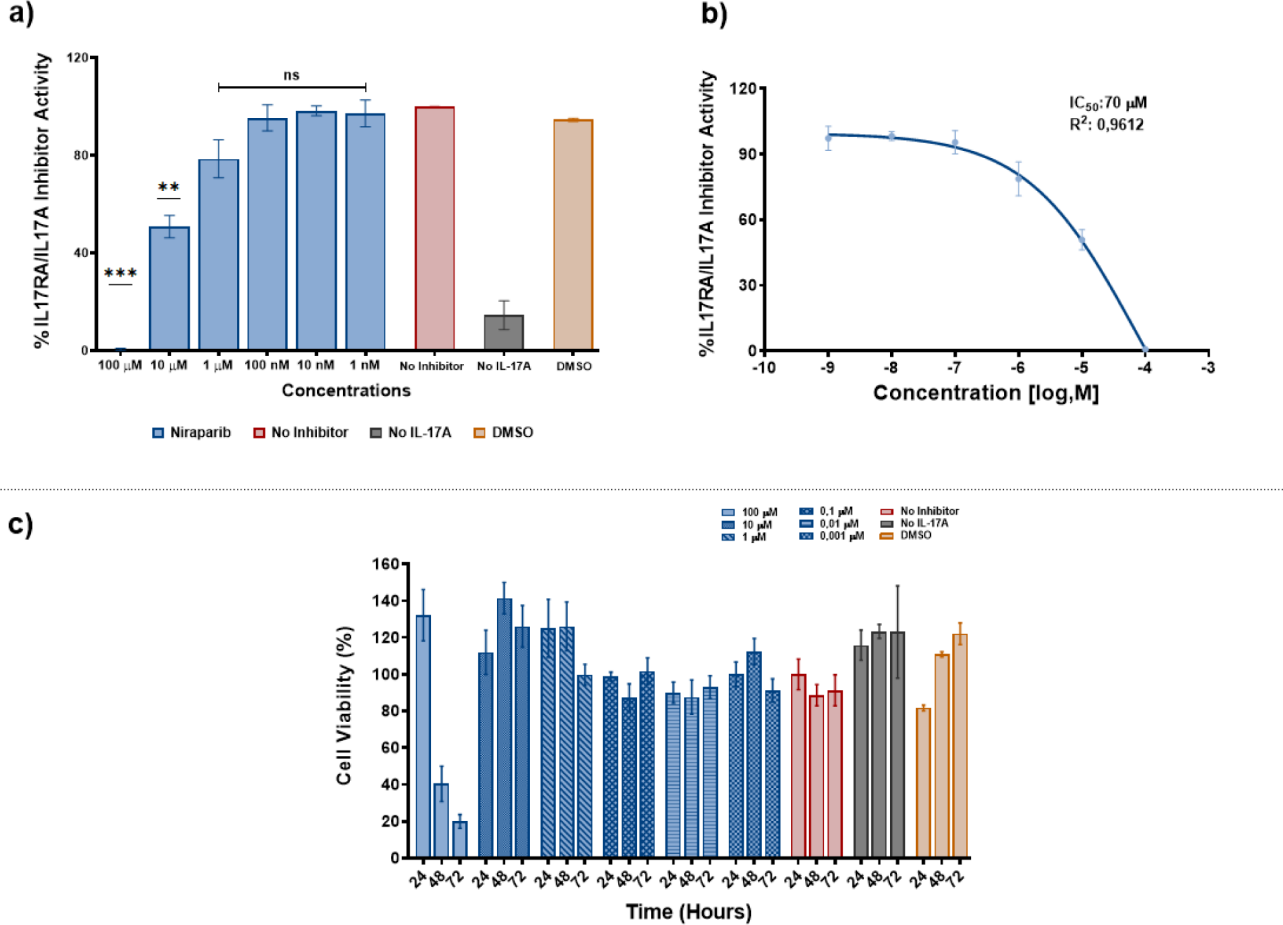
Reporter assay and MTT assays to evaluate inhibitory activity and toxicity of Niraparib. a) The graph illustrates the inhibitory effect of Niraparib on HEK-Blue IL-17 cells at various concentrations. The bars represent the IL-17A/IL-17RA interactions in the presence of the IL-17A ligand. Blue bars indicate the percentage of interactions with Niraparib, while the red bar shows ligand−receptor interactions without any inhibitors as a positive control. The gray bar (negative control) displays interactions that occur without IL-17A in the environment. The yellow bar, representing the DMSO control, demonstrates the effect of the solvent on the interaction. b) The line graph presents the calculated IC_50_ values corresponding to six different concentrations, ranging from 1 nM to 100 μM. c) MTT graphs illustrate the toxic effects of Niraparib at different concentrations on transgenic HEK293 cells. The red bars serve as the positive control to indicate cell viability. The yellow bars represent the DMSO control, showing the effect of the solvent on cells. The blue bars demonstrate the impact of Niraparib on cell viability across various concentrations.

### Niraparib Effectively Reverses Cuprizone-Induced Damage

The cuprizone mouse model was conducted to reveal the in vivo potential of Niraparib on demyelination. The cuprizone model was established by adding 0.4% cuprizone to mouse chow for 8 weeks to observe chronic demyelination (demyelination phase). After 8 weeks of cuprizone feeding, the cuprizone was removed from their diet, and the mice were fed normal chow for the following 8 weeks (remyelination phase). The healthy control group was fed normal chow for 16 weeks. The cuprizone control mice received only 1× PBS, while the treatment group received Niraparib after cuprizone removal. The degree of demyelination in the brain was evaluated through LFB-stained brain sections, which were analyzed at two time points to assess both demyelination and remyelination status. The extent of demyelination was assessed in terms of the integrity of the myelin sheaths in the corpus callosum region by three blind independent assessors. In addition to the integrity of the myelin sheaths, the color density in myelinated and demyelinated areas was compared. Thus, an increase in myelination levels was observed following CPZ removal (remyelination phase). Additionally, pale LFB-stained regions indicated excessive demyelination during the demyelination phase. Mice treated with Niraparib showed more remyelination compared to the control group at the remyelination phase (Figure 3b).

**Figure 3:**
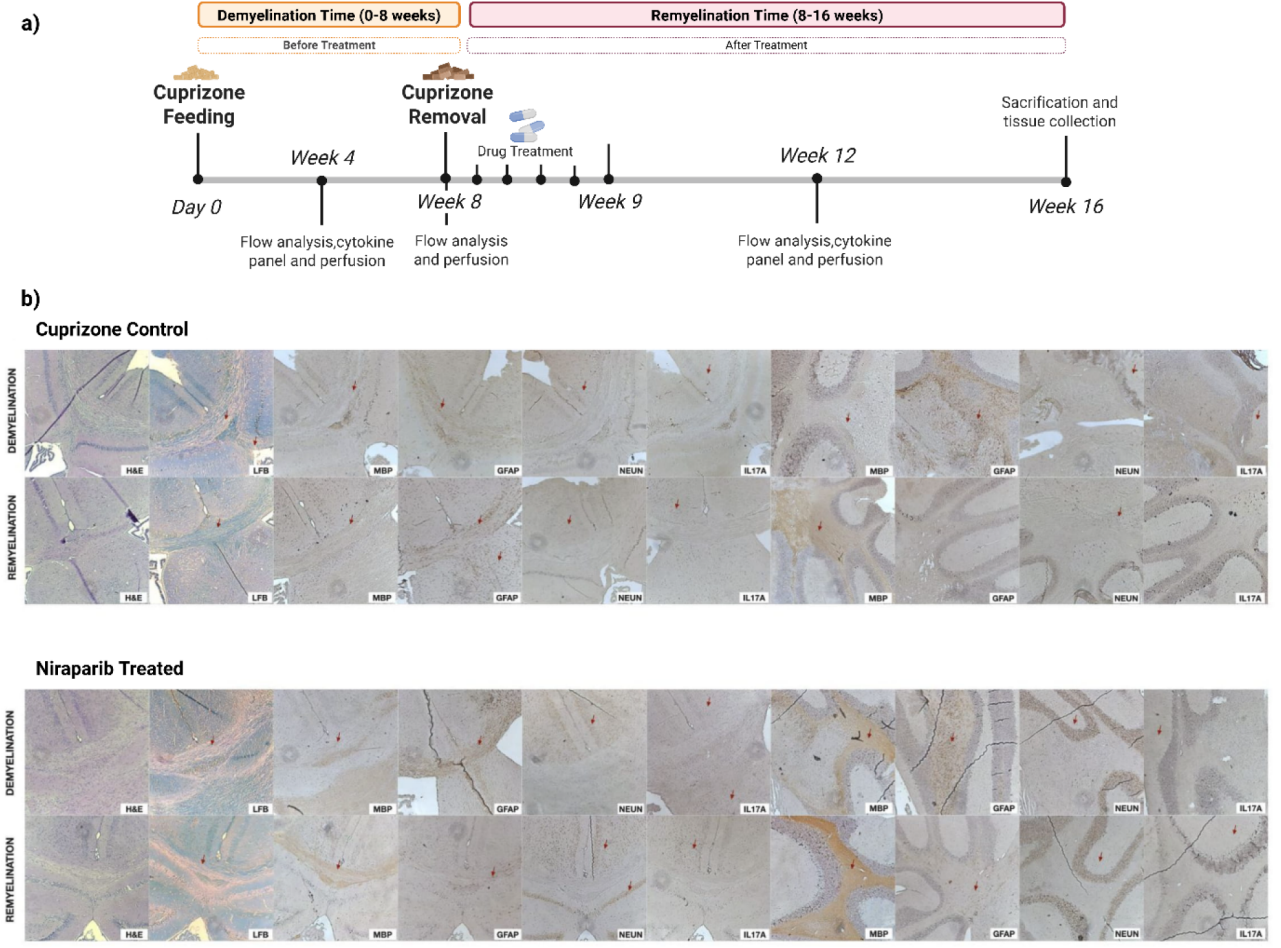
a) Summary of in vivo experimental design. The treatment group (*n* = 6) received 5 doses of Niraparib (2 mg/mL) following the 8 weeks of cuprizone treatment. Molecular markers of myelination and inflammation were observed by flow cytometry and histological staining (*n* = 1). b) Histochemistry analysis showed the myelination in corpus callosum of cuprizone mice group and Niraparib-treated group before and after treatment. LFB images were shown in 5× magnifications. MBP, GFAP, NeuN and IL-17A were shown 20× magnification. Red arrow shows the marker positive cells. MBP and GFAP were observed on the corpus callosum, while NeuN and IL-17A positive cells were observed cerebral cortex area.

In addition to LFB staining, MBP and GFAP staining were conducted in the corpus callosum to evaluate changes in myelin before and after treatment. The corpus callosum, the largest white matter structure in the brain, connects similar cortical regions across the two cerebral hemispheres. This area is essential for the transfer of sensory, cognitive, and motor information, making it a key focus in demyelination research. Myelin basic protein (MBP) immunostaining revealed loss of myelin in the mice fed the cuprizone diet at the demyelination time, which was strongly increased in both the CC and cerebellum of Niraparib-treated mice at the remyelination time (Figure 3b). An opposite demyelination trend was observed with GFAP immunostaining, as the GFAP marker indicates astrogliosis while MBP represents oligodendrocyte regeneration. The number of GFAP-positive cells increased during the demyelination stage in cuprizone-only treated mice. GFAP-positive cells seen in cuprizone-only fed mice at the time of demyelination decrease at remyelination time. When compared with the Niraparib-treated group, GFAP-positive cells are almost absent after Niraparib treatment. Furthermore, GFAP-positive cells were more abundant in the CC compared to the cerebellum; therefore, the decrease upon Niraparib treatment was more pronounced in the CC. These results demonstrate that Niraparib alters the myelin and affects cuprizone-induced demyelination.

Neuronal death in the cerebral cortex was examined using NeuN immunohistochemistry staining. NeuN staining serves as a marker for neuronal nuclei and was employed to evaluate neurons. Neuronal loss was observed in the cerebral cortex, indicating chronic demyelination. NeuN positive cells decreased during the demyelination stage, while the Niraparib-treated group displayed a notable increase during the remyelination stage in the CC, but no significant changes were observed in the cerebellum. IL-17A levels showed no significant differences between the demyelination and remyelination stages in cuprizone-only treated mice, while the number of IL-17A positive cells significantly decreased during the remyelination phase in both the CC and cerebellum. Overall, it can be concluded that Niraparib treatment induced recovery from cuprizone-induced demyelination (Figure 3b).

### Niraparib Treatment Induced Lymphocyte Enrichment and Activation in the Cuprizone-Treated Mice

Although the cuprizone model is primarily a nonautoimmune demyelination model, it is important to examine the peripheral immune system due to its impact on CNS inflammation and remyelination processes. This will facilitate comparison of the model with diseases such as MS and contribute to the development of new therapies targeting neuroinflammation. The immune cell subsets and cytokine profile in response to Niraparib treatment were investigated by evaluating the corresponding cell surface and intracellular proteins. The timeline was divided into three groups: pretreatment (0−8 weeks), early treatment (8−12 weeks), and late-treatment (12−16 weeks). The late treatment phase shows the development of the immune response over an extended period, while the change from pretreatment to early treatment indicates the immune response against cuprizone removal and Niraparib treatment. Furthermore, the presence and absence of T cells were investigated using CD3+, CD4+, and CD8a+ cell markers to analyze the density of inflammatory infiltrates associated with demyelination. As illustrated in Figure 4a, the level of CD3+ T lymphocytes was 36% before Niraparib treatment (in the pretreatment phase). However, Niraparib treatment increased CD3+ cells by 7% (*p* = 0.1) in the early treatment phase. Without treatment, the CD3+ level decreased by 5% after cuprizone removal (from pretreatment to early treatment). The difference between groups indicates a significant change in mature T cell levels (*p* = 0.1). The CD4+ level decreased by 13% from pretreatment to early treatment in the cuprizone-only treated group but then returned to nearly the same level as pretreatment during the late stage (*p* = 0.1). CD4+ T helper cells were elevated and significantly differed in the early stage of Niraparib-treated mice (*p* = 0.07); however, the decrease in the late stage was insignificant. To evaluate the recognition of MHC class I molecules for killing target cells by inducing apoptosis, CD8a+ T cells were analyzed. The level of cytotoxic CD8a+ T cells varied between untreated control mice and cuprizone-treated groups in the pretreatment phase. During the early treatment phase, the cell population significantly decreased (*p* = 0.001) in the cuprizone-treated group, while the Niraparib-treated mice group showed an equivalent increase during this stage. The cell levels reached similar amounts at the late-treatment phase; however, the Niraparib group exhibited a significant decrease (*p* = 0.01), while the cuprizone group demonstrated an increase in the late stage (Figure 4a). In most EAE models, CD4+ T cells dominate the infiltration of inflammatory cells. However, the majority of infiltrated T cells in MS lesions consist of CD8+ T cells.^31^ In this study, there was no T cell dominance. Moreover, the activated helper and cytotoxic T cells were evaluated based on the number of CD3+CD4+/CD8a+ triple-positive cells. The active cell population decreased after cuprizone removal (pretreatment phase); however, there was a significant increase in the cuprizone-only treated mice (*p* = 0.03) at the late stage. In the Niraparib-treated group, the level remained unchanged at the late stage; however, a significant increase was observed from pre- to early treatment duration (*p* = 0.02).

CD25 plays a critical role in immune system function as part of the IL-2 receptor. It is also an important marker for regulatory T cells (Tregs) that prevent the development of immune-mediated diseases by inhibiting the pathological activation of immune cells. It decreased to 2% of regulatory T cells (CD25+) in cuprizone-treated mice after cuprizone was removed from their diet (from pretreatment to early treatment), while the level of cells remained almost the same before and after Niraparib treatment. It was observed that there was no significant change in regulatory T cells in both groups. As another regulatory T cell marker, the CD8b+/CD25+ T cells are regulatory cells that have a role in the activation of both cytotoxic T cells and modulating the immune response by cytokine secretion. In the late period, the cell level in the cuprizone-only treated group remained the same as the pretreatment time. However, there was a significant drop after cuprizone removal (at the early treatment time, *p* = 0.09), and then the cell population elevated to the initial level. On the other hand, Niraparib treatment increased 8% of the cell density after drug treatment (early treatment time, *p* = 0.02), and the cell level remained the same. The transcription factor, FoxP3, increased more than 10% from pretreatment to the early treatment in the CPZ-induced demyelination control mice (*p* = 0.02). Also, the comparison between Niraparib treated mice and untreated mice showed the significant increase in the regulatory cells of the mice treated with Niraparib (*p* = 0.009). To analyze effective regulatory T cells CD4+/CD25+/FoxP3+ levels were analyzed. CD4+/CD25+/FoxP3+ typically identifies regulatory T cells (Tregs) and CD25+/Foxp3+ double-positive staining indicates mature Tregs (Figure S3). Ly6C/Ly6G is cell surface glycoproteins expressed on monocytes, macrophages (Ly6C), and neutrophils (Ly6G) and involved in various immune functions, including inflammation and tissue repair. It was found that Ly6C/Ly6G+ cells of untreated cuprizone mice slightly increased in the late stage. Niraparib-treated mice showed a significant drop at early treatment time (*p* = 0.02), then a 6% increased cell population was seen in the late stage (*p* = 0.1) (Figure 4b).

**Figure 4:**
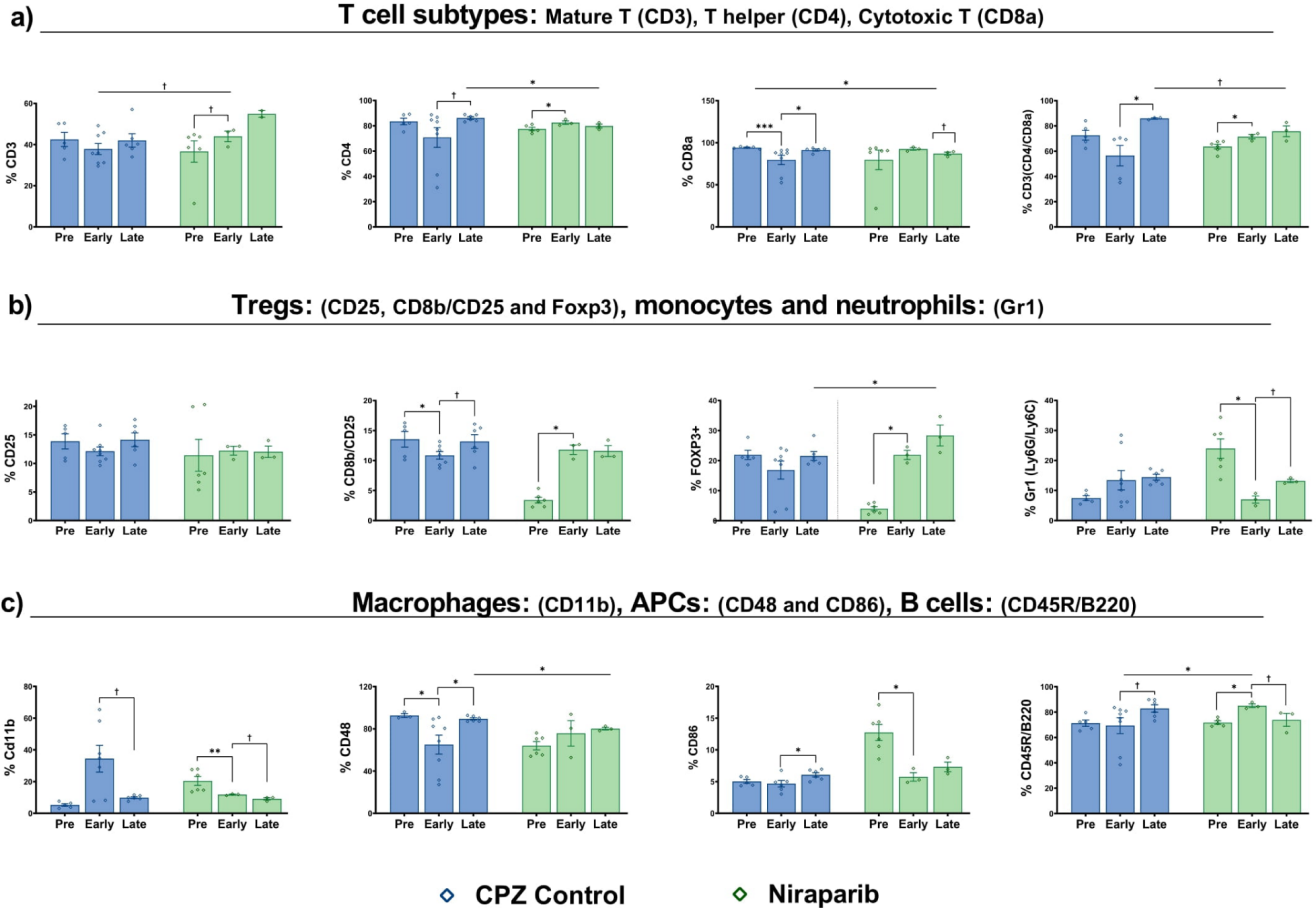
Immune profiling of cuprizone treated and Niraparib drug treated mice in early and late treatment. Data was derived from blood. Blue and green bars indicate cuprizone-treated (untreated) and Niraparib treated mice groups, respectively. *X*-axis represent the weeks that include receiving treatment (early: 0−8 weeks and late 8−16 weeks). Differences were determined using Kruskal−Wallis Test followed by Mann−Whitney test to compare the medians of two independent groups. The following symbols are used to indicate the level of significance: † for *p* < 0.1, * for *p* < 0.05, ** for *p* < 0.01, *** for *p* < 0.001.

CD11b is primarily expressed in myeloid cells, including macrophages, neutrophils, and dendritic cells. It plays a role in cell adhesion as part of the immune response. The level of CD11b+ cells was higher in cuprizone-treated mice during the early stage, whereas all three groups exhibited nearly the same cell density in the late stage of the model. The level of macrophages surged in the early stage of cuprizone-treated mice; however, this increase was not evaluated statistically. The decrease in CD11b+ cells from the early to late treatment stage in cuprizone-only treated mice was significant (24%). Niraparib treatment resulted in a gradual decrease of CD11b+ levels. A significant drop was observed (*p* = 0.02) at the early stage, while a 2% reduction was noted after treatment (*p* = 0.1). CD48 is a glycoprotein that is expressed on various immune cells, and it promotes the organization of the immune synapse and the costimulation of T cells by binding to CD2 on T cells of antigen-presenting cells (APCs). Shortly, this marker indicates the cell−cell communications and organizing the immune response. After cuprizone removal, the APC level was lowered at early treatment time. From the early treatment time to late-treatment time, the level of APCs increased % by 30 in cuprizone-treated groups (*p* = 0.02). However, Niraparib treatment suppressed the decrease at the early stage and the increase of CD48+ cells low in the late stage (Figure 4c). CD86 is a costimulatory molecule found on antigen-presenting cells (APCs), including dendritic cells, macrophages, and mature B cells, and it plays a role in T cell activation. At the early treatment time, no change was observed in the cuprizone-only treated group, while a 7% decrease (*p* = 0.02) was noted in the Niraparib-treated mice. The level of CD86+ cells increased similarly in both cuprizone-only treated (*p* = 0.04) and Niraparib-treated mice (*p* = 0.05) from early treatment to late treatment. CD45R/B220 expression is primarily associated with B lymphocytes. From pretreatment to early treatment, there was no significant drop in the cuprizone group, while a 14% increase was seen in the Niraparib-treated group (*p* = 0.03). Additionally, CD45R/B220+ cells decreased in Niraparib-treated mice at the late treatment time (*p* = 0.1), while the untreated cuprizone group experienced a similar decrease (*p* = 0.1) (Figure 4c). As a result of considering the immune system cell markers, one can conclude that Niraparib treatment changed the levels of lymphocyte markers during the early stages of drug treatment, specifically after 8 weeks of cuprizone administration. These cells were consistently present in the late stages of the cuprizone mice model following Niraparib treatment.

### Niraparib Suppressed the IL-17 Immune Response by Regulating Pro- and Anti-Inflammatory Cytokines

The proliferation of microglial cells is one of the key points in cuprizone-induced demyelination. This proliferation causes a significant microglial/macrophage response, leading to the production of cytokines such as TNF-α by these cells during demyelination. In light of the involvement of inflammatory cytokines in the myelination process, a cytokine panel was assessed. Cytokine levels were measured at the fourth and 14th weeks of the CPZ model; however, the timeline shown in the graphs indicates 0−8 weeks (before treatment) and 8−16 weeks (after treatment). The IL-23 cytokine plays a crucial role in maintaining and producing T helper and Th 17 cells and is secreted by activated T cells. The level of IL-23 dropped significantly after Niraparib treatment (*p* = 0. 05), while cuprizone-treated mice showed no significant change. IL-17A is the primary pro-inflammatory cytokine produced by IL-23 and other cytokines to initiate inflammatory responses. CPZ-treated mice demonstrated a significant decrease in IL-17A levels after the removal of cuprizone (*p* = 0.1), whereas the Niraparib group exhibited an increase following treatment (the significance was not calculated). IFN-γ plays a role in modulating immunity. The level of IFN-γ decreased in cuprizone-treated mice, while it significantly increased after Niraparib treatment (*p* = 0.1). MCP-1 acts as a chemoattractant to draw cells to inflamed areas. The cuprizone-treated group showed a gradual decrease in MCP-1 cytokine levels (*p* = 0.1), while there was almost no change in the Niraparib-treated group. IL-10 is an anti-inflammatory cytokine that increased in the cuprizone treatment group (*p* = 0.1) and in the Niraparib-treated group after cuprizone removal. IL-6 is both a pro-inflammatory and anti-inflammatory cytokine that triggers the production of IL-10. It was found that IL-6 levels significantly increased in the Niraparib group (*p* = 0.1), which is consistent with IL-10 production (*p* = 0.1). IL-27 is another pro-inflammatory and anti-inflammatory cytokine that regulates the IL-17 response of immune cells. It has been shown that IL-27 significantly decreased after treatment in both cuprizone and Niraparib-treated mice (*p* = 0.05). IL-1 α, IL-1 β, IL-12 p 70, TNF-α, IFN-β, and GM-CSF cytokines showed no significant change before and after treatment in the Niraparib and cuprizone groups (Figure 5). Cytokine assessment demonstrated that Niraparib treatment upon 8 weeks of cuprizone administration suppressed pro-inflammatory cytokines and increased the anti-inflammatory cytokines.

**Figure 5:**
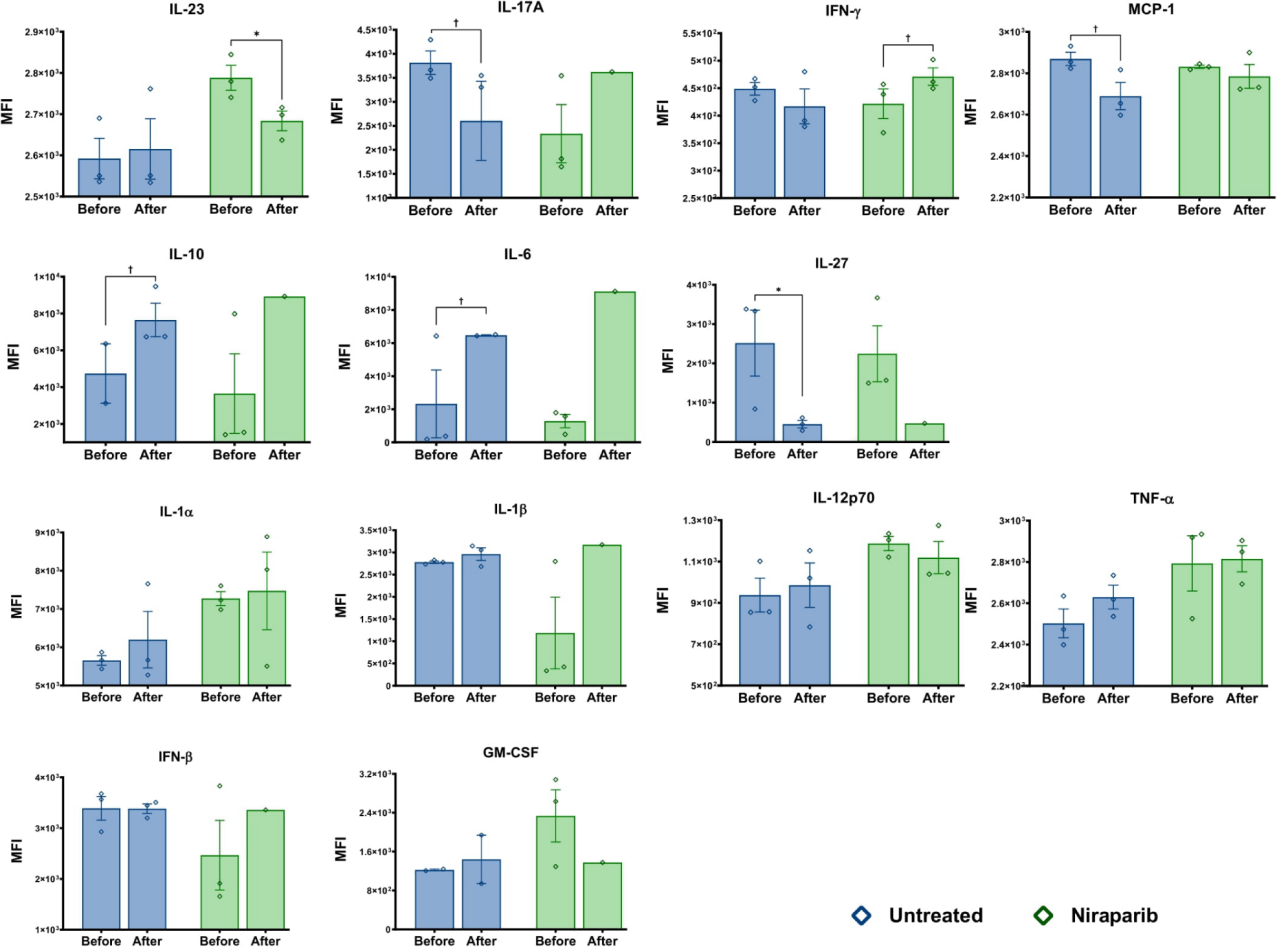
Cytokine levels from the plasma samples of cuprizone treated and drug-treated mice. *Y*-axis indicates MFI, which stands for “Means Florescence Intensity”. *X*-axis represents weeks which was separated before and after treatment (0−8 weeks: before treatment and 8−16 week: after treatment). Differences were determined using Kruskal−Wallis Test followed by Mann−Whitney test to compare the mediand of two independent groups. The following symbols are used to indicate the level of significance: † for *p* < 0.1, * for *p* < 0.05, ** for *p* < 0.01, *** for *p* < 0.001.

## Discussion

PARP is a family of 17 human enzymes that coordinate DNA repair, transcriptional regulation, cell cycle, oncogene activity, and mitochondrial function through an ADP-ribosylation post-translational modification called PARylation. PARP-1 inhibitors are successful cancer therapies that disrupt the DNA repair mechanism, leading to accumulation of DNA damage and ultimately cell death. The common mechanisms behind cancer and demyelinating diseases, alongside the effectiveness of anticancer drugs as repurposed treatments, highlight the potential of using PARP-1 inhibitors for treating demyelinating diseases. In addition, PARP-1 inhibitors exhibit remarkable neuroprotective effects in demyelinating disorders, including MS, which is considered the archetypal demyelinating disease.^29^ PARP-1 is a nuclear DNA-binding protein belonging to a family of more than 17 members that is activated by DNA damage. It catalyzes the binding of ADP-ribose to target proteins and acts as a component of enhancer/promoter regulatory complexes. It plays a role in regulating DNA repair and maintenance of genomic integrity. Numerous studies have also implicated PARP-1 in the regulation of the inflammatory response. These studies have laid the foundation for the potential clinical applications of PARP inhibitors.^32^ There are many reports revealing the imbalance of Treg and Th17 cells in lung diseases. A related study examines the effect of PARP-1 on Th17/Treg imbalance and the potential mechanism in premature rats with acute respiratory distress syndrome (ARDS). According to the study, downregulation of PARP-1 reduces the expression level of IL-6 and consequently suppresses the imbalance of Th17 and Treg cells.^33^ One study on the relationship between PARP-1 and IL-17A examined the effects of Olaparib in oral squamous cell carcinoma (OSCC) patients. Olaparib has shown promising potential in enhancing the effectiveness of radiation therapy and preventing metastasis. This is largely due to its ability to inhibit IL-17A-dependent signaling pathways, showcasing its dual role as both a radiosensitizer and an agent against cancer spread.^34^ Rheumatoid arthritis (RA) is one of the major autoimmune diseases with global prevalence. In a study conducted in adjuvant induced arthritis (AIA) mice, a model of rheumatoid arthritis (RA), the PARP-1 inhibitor 5-aminoisoquinolinone (5-AIQ) reduced disease severity, lowered arthritis scores, and suppressed the levels of inflammatory mediators. 5-AIQ treatment decreased IL-17A, NF-kB p65, and GITR-expressing cells, while increasing regulatory T (Treg) cells, IkB-α levels, and anti-inflammatory mediators. These results indicate that PARP1 inhibitors may serve as anti-inflammatory and antiarthritic agents for conditions such as rheumatoid arthritis.^32^

Targeting proinflammatory cytokines, such as TNFα, represents a clinically validated therapeutic strategy across a spectrum of autoimmune disorders, including psoriasis, Crohn’s disease, psoriatic arthritis, ankylosing spondylitis, rheumatoid arthritis, and ulcerative colitis. Emerging targets within the IL-17-Th17 axis present opportunities to enhance the efficacy of current treatments for inflammatory conditions. Ustekinumab, an inhibitor of IL-12 and IL-23, modulates multiple Th cell pathways, including Th17, and has received regulatory approval for psoriasis and psoriatic arthritis.^35^ Contemporary therapeutic advancements encompass numerous investigational agents targeting various components of the IL-17-Th17 pathway for diverse inflammatory states. Some of these candidates, such as the cytokine IL-17 itself, its receptor IL-17RA, and the p19 subunit of IL-23 (essential for Th17 cell differentiation), are under active investigation. Secukinumab and ixekizumab are monoclonal antibodies that specifically neutralize IL-17A.^35^ Monoclonal antibody (mAb) therapies are now a cornerstone in the management of many chronic inflammatory diseases.^36^ Interleukin 17 mAb inhibitors, including ixekizumab, secukinumab, and brodalumab, have demonstrated clinical efficacy in the biological treatment of psoriasis, psoriatic arthritis (PA), ankylosing spondylitis (AS), and rheumatoid arthritis (RA), while also playing a role in mediating the immune response against bacteria and fungi.^37^ Secukinumab, ixekizumab (both targeting IL-17A), and brodalumab (targeting the IL-17 receptor) constitute the primary mAbs utilized in autoimmune and inflammatory disease therapeutics; of these, secukinumab has received approval from the United States Food and Drug Administration (FDA) for the treatment of plaque psoriasis, PA, AS, and nonradiographic axial spondyloarthritis. Anti-IL-17A and anti-IL-17RA mAbs can elicit cell killing through distinct mechanisms. They form antigen−antibody complexes (IL-17A/anti-IL-17A Ab and IL-17RA/anti-IL-17Ab) via binding to the FAB region, thereby inhibiting the interaction of IL-17 with its receptor. This blockade consequently suppresses the production of proinflammatory cytokines and chemokines implicated in the pathogenesis of several diseases.^38,39^

While injectable mAbs exhibit a favorable safety and efficacy profile, the exploration of orally bioavailable small molecules with comparable efficacy and safety represents a significant area of research for alternative treatment strategies. In contrast to anti-IL-17A mAb, the first oral drug approved for MS was fingolimod, which modulates the S1P receptor, thereby preventing the egress of specific activated T cells from lymph nodes and their subsequent entry into the central nervous system. However, clinical investigations have revealed concerns regarding the adverse effects of cardiac effects of this drug, as well as an observed association with the development of certain malignancies and an increased susceptibility to infections. Fingolimod, teriflunomide, and alemtuzumab are established therapeutic agents for MS, but their clinical utility is tempered by serious side effects, including cardiovascular complications.^40,41^ Currently, there is no curative therapy for MS; however, immunomodulatory drugs that aid in disease management and slow its progression are preferred in MS treatment due to their safety profiles. Initial evidence supporting the efficacy of IL-17 inhibition in reducing lesion activity in MS patients emerging from studies evaluating secukinumab. Concurrently, the safety profile of secukinumab has been observed to be generally favorable in patients. The development of CJM112, a novel anti-IL-17 human mAb with potentially enhanced efficacy in treating MS patients, led to the discontinuation of clinical trials involving secukinumab in this specific indication.^42^ Given the established safety and efficacy of injectable mAbs, the pursuit of orally administered small molecules with comparable therapeutic profiles is paramount. However, modulating large protein−protein interactions (PPIs) with small molecules presents a considerable challenge, as extensively reviewed in the literature concerning PPI druggability.^43^ As highlighted by Andrews et al. (2022), initial indications that small molecules could modulate IL-17A arose in 2013 with a patent application from Ensemble Therapeutics, which demonstrated the efficacy of large macrocyclic molecules identified through DNA-encoded molecule library screening.^44^ Ensemble 159, a specific small molecule detailed in their publication, exhibited cellular activity by specifically binding to IL-17A and modulating its response in human keratinocytes and fibroblasts. Their study delineates the research leading to the identification of a small molecule protein−protein interaction modulator (PPIm) as a candidate for clinical development. Protein crystallography provides critical insights into the key binding interactions between small molecule ligands and the IL-17A dimer. Structural information on IL-17A is particularly crucial for targeting PPIs, as it facilitates rational design strategies to optimize ligand binding modes and, consequently, their potency.^44^ Numerous therapeutic modalities, including small molecule drugs (SMDs) and biologics such as mAbs, have been approved for the treatment of neurological diseases. While both are considered targeted therapies, they exhibit distinct characteristics that influence their clinical applications. SMDs are relatively small (∼0.5 kDa) and chemically simple, whereas mAbs are large (∼150 kDa), complex proteins derived from living cells, requiring intricate and quality-controlled production processes to ensure batch consistency. SMDs typically necessitate daily administration, often orally, whereas mAbs, due to their extended half-lives, may be administered at monthly intervals. The smaller molecular weight of SMDs offers a significant advantage in their ability to traverse the BBB, a critical factor in the treatment of neurological disorders.^45^

Previous investigations reported that macrocyclic structures (PDB: 5HI3, 5HI4, 5HI5) binding to the N-terminal region of homodimeric IL-17A also interact with the same site as the IL-17 receptor.^46^ Another study, introduced a novel class of inhibitor molecules that interact with IL-17A at the C-terminal binding site.^47^ These molecules characterized using NMR-based Surface Plasmon Resonance fragment screening (PDB: 8DYI, 8DYH, 8DYG, 8DYF), target another critical region for IL-17A dimer interaction with cell surface receptors. While X-ray crystallographic studies have elucidated the binding of macrocyclic structures to the N-terminal region of IL-17A, these molecules have not yet progressed to clinical trials. Similarly, macrocyclic molecules interacting with the C-terminal region of the IL-17A dimer have not been evaluated in clinical trials, although FRET analyses indicated their ability to block binding to IL-17A with an IC_50_ of approximately 9 nM.^47^ As detailed in their study, these C-terminal interacting molecules were designed using NMR-based fragment screening, revealing a previously unreported binding mode. Additional molecules binding to other sites on the IL-17A homodimer have been reported. However, for the C-terminal molecules, achieving adequate solubility and permeability remains a significant challenge. A report from Leo Pharma indicated that a macrocyclic structure identified by Pfizer exhibited favorable oral pharmacokinetic properties. UCB and other companies have proposed alternative strategies for small molecules targeting IL-17A in their patent applications.^47^ Building upon the understanding of inflammatory mechanisms and therapeutic targets discussed above, it is noteworthy that PARP-1, a nuclear enzyme, also emerges as a significant player in chronic inflammation associated with various inflammatory-mediated pathologies and tumors. PARP-1 plays a significant role in chronic inflammation associated with various inflammatory-mediated pathologies and tumors. Reports indicate that PARP-1 knockout mice or mice treated with PARP-1 inhibitors are resistant to different types of inflammation, such as lipopolysaccharide-induced septic shock.^29,48,49^ The pro-inflammatory effect of PARP-1 has not been influenced by immune cells; rather, astrocytes, endothelial cells, and fibroblasts also contribute to the inflammatory process. Additionally, PARP-1 is expressed by cells in the CNS and is involved in various biological processes, such as cell differentiation, maturation, memory formation, and the regulation of cholinergic and glutamatergic signaling. When examining the relationship between PARP-1 and oligodendrocytes, it is indicated that PARP-1 serves as a positive regulator of oligodendrocyte differentiation and maturation during CNS development. Oligodendrocytes are susceptible to oxidative stress, inflammation, and DNA damage. Injury to these cells ultimately leads to myelin loss around neuronal axons and disorders in synaptic transmission and axonal function. Consequently, neurological diseases such as MS and leukodystrophy arise. Recent publications have demonstrated that oligodendrocyte density is decreased in PARP-1 knockout mice, leading to hypomyelination in the corpus callosum.^29,50^ These studies suggest that enhancing PARP-1 therapeutically may be a viable strategy for inducing remyelination in demyelinating diseases. However, some research indicates that PARP-1 prevents the death of oligodendrocytes in conditions like MS and ischemia. Additionally, it has been reported that the role of PARP-1 in oligodendrocytes may depend on the cell’s age and a specific stage of cell differentiation. Despite this, studies support a direct role for PARP-1 in oligodendrocyte differentiation, survival, and myelination status. In experiments using allergic encephalomyelitis (EAE), PAR, a marker of PARP-1 activation, was found to accumulate in oligodendrocytes, microglia, neurons, and in astrocytes surrounding demyelinating plaques. In the EAE model, selective PARP-1 inhibitors PJ34 and INH2BP reduce the expression of inflammatory cytokines TNF-α, IL-1β, interferon-γ (IFN-γ), interleukin-2 (IL-2), and iNOS in the spinal cord, thereby reducing disease progression and improving symptoms.^29,51,52^ Additionally, various studies indicate that PARP-1 inhibitors diminish the infiltration of immune cells by altering the BBB and inhibiting PARP-1 activity in monocytes.^29^

PARP-1 can increase IL-17A production by activating the NF-κB signaling pathway, which can lead to an increase in inflammation in autoimmune diseases such as MS. PARP-1 inhibitors can reduce inflammation by suppressing IL-17A expression, the use of niraparib as an IL-17A inhibitor may be a promising approach in the treatment of autoimmune diseases by offering a new anti-inflammatory strategy. Our study identifies a potential compound, Niraparib, as an IL-17A inhibitor through Binary-QSAR model followed by *in vitro* and *in vivo* studies. Niraparib is a small molecule inhibitor primarily recognized for its role as a poly­(ADP-ribose) polymerase (PARP) inhibitor in treating ovarian and breast cancers.^26,27^ Its main mechanism of action involves the inhibition of DNA repair process that leads to the accumulation of DNA damage in cancer cells, which triggers cell death. Niraparib has been shown to have an immunomodulatory role by activating interferon pathways and increasing the infiltration of CD4+ and CD8+ immune cells. PARP inhibition increases peritoneal CD8+ and natural killer cell numbers and functions but also increases FoxP3+, CD4+, and regulatory T cells.^53^ In addition to their critical roles in anticancer activity and immunomodulation, PARP inhibitors hold significant promises for enhancing the immune response, offering a compelling avenue for advancing treatment strategies. The direct interaction between Niraparib and IL-17A remains unclear. We demonstrated for the first time that Niraparib has the potential to inhibit the binding of IL-17A to its receptor, IL-17RA.

This study suggests that Niraparib may act as a therapeutic anti-inflammatory agent for MS, with a particular emphasis on the IL-17A/IL-17RA interaction. The initial binary QSAR-based predictions for Niraparib’s anti-inflammatory TAV, calculated using the Metacore/Metadrug platform, yielded a value of 0.56. This indicates that Niraparib has a Tanimoto coefficient comparable to that of known anti-inflammatory agents. This suggests its potential as an inflammation modulator by binding to interleukins, the primary agents of inflammation. We then used physic-based molecular simulations to predict the binding mode of Niraparib to the aforementioned binding regions of the IL-17A/IL-17RA interface and estimate the relative binding affinity. Our workflow composed of binding site identification, molecular docking and MD simulations can shed light on the binding mode of Niraparib induced inhibition and the molecular mechanism involved behind it. For this purpose, we have first identified the crucial binding sites between IL-17A/IL-17RA which were Region I (S64 and R101) and Region II (D42, R55, V65, and W67) in the N-termini, as well as Region III (Y85 and H86) and Region IV (I127, V128, H129, H130, and V131) in the C-termini and used a grid-based approach to dock Niraparib to these specific regions. In this context, these regions were targeted with Niraparib, hypothesizing that this would lead to the dissociation of the complex. We have decided to conduct Niraparib complex MD simulations at the C-terminal Region IV, since it had the lowest docking score of −6.58 kcal/mol and Niraparib had multiple hydrogen bonds, electrostatic and van der Waals interactions with both A, B and C chains. Furthermore, the C-termini of IL-17A undergoes a conformational change upon binding to IL-17RA and shifts from disordered to ordered in the vicinity of Region IV.^30^ Due to this reorganization governed by loss in entropy, we hypothesized that Niraparib may easily disrupt the binding of the C-termini of IL-17A to IL-17RA and mainly focused on this area specifically. Furthermore, the interactions between the C-terminal 127−131 residues of IL-17A and IL-17RA is specific to IL-17A and IL-17F, but not other ILs. Our findings indicate similar results to experimental reports. Niraparib most significantly interacts with the backbone carbonyl of S40 of chain B with hydrogen bonding through its carboxamide end and stabilizes itself inside the binding pocket. The rotatable bond holding the piperidine moiety is stabilized through polar interactions with E155 on chain C and hydrophobic interactions with L264 on chain C and water-mediated hydrogen bonding with the side chain amine of V128. Altogether, the thermodynamic evaluation of Niraparib at Region IV indicates that this may be the molecular mechanism of Niraparib-based disruption of an already formed IL-17A/IL-17RA complex.

The CPZ model is a widely used experimental model for studying CNS demyelination to understand the pathology of MS. In the CPZ mouse model, demyelination is highly reproducible in the CC region and cerebellum region.^23^ Approximately 6 weeks of 0.2% cuprizone treatment leads to acute demyelination in the CC region, while extending the treatment to 12 weeks or increasing the amount to 0.4% leads to chronic demyelination^17^ Following acute demyelination, spontaneous remyelination begins immediately once cuprizone is eliminated from the diet. However, when chronic demyelination develops, remyelination is restricted despite cuprizone withdrawal.^54–56^ Anti-inflammatory agents, neuroprotective agents or agents promoting remyelination like retinoic acid derivatives are notable molecules to treat cuprizone-induced demyelination.^19,57^ CPZ mouse model is also used for evaluating microglia activation and neuroinflammation in CNS, however damage in the BBB led to lymphocyte infiltration to enhance the demyelination. Therefore, the peripheric inflammation influences the progress of disease. Apart from the lymphocytes, inflammatory cytokines could affect inflammation on CNS. While the CPZ model was initially thought to be T cell independent, recent studies suggest that peripheral immune responses influence disease progression and recovery. Peripheral immune cell regulation influences microglial activation, inflammatory signaling, and CNS repair mechanisms in the CPZ model.^19^ Targeting peripheral immune pathways, such as IL-17A inhibition, may offer therapeutic strategies to enhance remyelination and reduce neuroinflammation in MS. Various studies have been examined to understand the effects of changes in the regulation of peripheral immune cells on the CPZ model. In a study that involved suppressing the peripheral immune system, it was found that the central nervous system mechanism was notably limited in the CPZ model. The research demonstrated that CPZ caused atrophy in peripheral immune organs, such as the spleen and thymus, and led to a reduction in T-cell levels.^58^ Another study indicates that while the CPZ model was initially thought to be independent of T cells, it has limitations in assessing the immune system’s effects.^19^ CPZ-induced demyelination triggers the migration of CD8+ T cells, particularly those with a cytotoxic phenotype, into the central nervous system.^31^ In addition, another study reported that atrophy in peripheral immune organs and suppression of T-cell levels was observed in CPZ-fed mice, which poses limitations in the CPZ model in examining the effects of the immune system in demyelinating diseases.^22^ Finally, it is shown that oligodendroglial damage in the CPZ model triggers the migration of peripheral immune cells into the central nervous system.^59^ These studies provide important findings in understanding how the regulation of immune cells in the CPZ model shapes central nervous system pathology. Considering these, the CPZ model was chosen in this study because it effectively mimics key pathologic features of MS as well as allowing for the investigation of both CNS and peripheral immune mechanism. Also, it provides a valuable platform for evaluating potential therapeutic strategies targeting neuroinflammation and remyelination. Although CPZ model has many advantages to study for neurodegeneration as well as myelin integrity, it has some limitations. One of the restrictions is that the evaluation of motor functions in cuprizone-induced demyelination in contrast to the EAE model.^60,61^ Another limitation of the CPZ model is that it a is a toxin-induced model rather than autoimmune model like EAE. However, there are many studies, mentioned previously, this model can show the change of peripheral immunity against cuprizone-intoxication. Niraparib makes changes in Th17 cell-induced inflammation. Notably, the model has been shown to recapitulate key aspects of the immune-mediated pathology observed in human MS, including the infiltration and activation of immune cells, such as microglia and T cells which contribute to the damage of myelin-producing oligodendrocytes.^62^ These findings reveal the decrease in the demyelination and suggest that screening molecules based on IL-17A structure could serve as a prototype for repurposing the drugs targeting IL-17A-mediated inflammatory diseases.

Molecular antibodies and experimental small molecules have been developed to modulate the IL-17A cytokine. Secukinumab and Ixekizumab bind directly to IL-17A, inhibiting its activity, while Brodalumab blocks the IL-17 receptor and inhibits the signaling pathway.^63^ In a previous study, it was shown that cuprizone-fed mice with IL-17A overexpression exhibited enhanced microglial activation and astrocytic responses.^9,10,18,64^ Remyelination begins as soon as cuprizone is removed from the diet, and it can be monitored by visualizing myelination using GFAP, LFB, and other IHC staining methods.^19,55,65^ LFB, GFAP, MBP, and NeuN staining revealed decreased demyelination following the removal of cuprizone. It was also observed that the recovery from demyelination correlates with MBP re-expression.^20^ Secukinumab demonstrated neuroprotection by promoting remyelination through an increase in the level of MBP in the corpus callosum, hippocampus, and cortex of the CPZ model.^8^ Consistent with this, the MBP staining results indicated the induction of demyelination due to damage to oligodendrocytes when retreating demyelination with Niraparib. The histopathological examination of astrocytosis in cuprizone-induced demyelination was assessed using the GFAP marker. Astrocytes are responsible for neuronal functions, the integrity of the BBB, and brain homeostasis.^8,66^ Typically, cuprizone increases GFAP expression in the hippocampus and brain cortex during demyelination.^66^ However, it was shown that mice treated with Secukinumab had decreased GFAP levels^8^ Niraparib treatment demonstrated similar changes in the brains of mice experiencing demyelination. In addition to GFAP and MBP proteins, NeuN was also utilized to show the extent of remyelination following Niraparib treatment. IL-17A inhibitors may have neuroprotective effects in the CPZ model as Niraparib reversed cuprizone-induced demyelination^8^ Furthermore, the role of IL-17 in activating microglia and astrocytes was reported in cuprizone-induced demyelination, indicating that IL-17 levels increased in the hippocampus and brain cortex of cuprizone-treated mice, underscoring the significance of IL-17A during demyelination.^67^ Nonetheless, Niraparib reduced the level of IL-17A in the cortex compared to the cerebellum, which showed consistent results with the remyelination observed following Secukinumab treatment.^8^ Considering the role of IL-17A in the activation of microglia and astrocytes that contribute to the pathogenesis of cuprizone-induced demyelination, it can be concluded that inhibiting IL-17A activity with Niraparib may provide protective effects against neuroinflammation and support the remyelination process by inhibiting the inflammatory response.

The level and activity of T, B and plasma cells are associated with axonal damage and neurodegeneration in MS progression.^68^ Axonal injury is primarily driven by inflammation in MS; however, it is important to note that this does not imply that immune cells directly cause acute axonal injury. Instead, it suggests that the degeneration of axons may serve as a trigger for the recruitment of immune cells.^31,68^ Conflicting results exist about the presence of T cells in CPZ model, while MS patients showed increased intracranial immune cell types in the brain.^31,58,69–72^ The CD3+ T lymphocytes in MS animal models were reported to be changing.^3,73–76^ A previous study showed that regulated peripheral immune cells’ recruitment was presented on the demyelination site on the brain of cuprizone-treated mice that modulated inflammatory responses. On the other hand, IL-17 mediated signaling induced CD3+ T cells to be activated.^31^ Further, regulatory T cells decreased the proinflammatory and cytotoxic effect of Th1, Th17 and T subtypes by differentiating oligodendrocytes and promoting the myelin repair.^31,75^ CD3+ T cells function by activating cytotoxic T cells and T helper cells.^23^ In other studies, CD3+ T level decreased after the 10th week in mice fed with cuprizone for 8 weeks^23^ while Niraparib reduced the increased CD3+ T cells over time. CD4+ and CD8+ lymphocytes are recruited into the CNS and trigger neurodegeneration. Specifically, cytotoxic CD8+ cells are predominantly present in the cuprizone-induced demyelination regions.^57^ This reflects a similar ratio of CD4+/CD8+ in human MS pathology; however, it differs from the EAE model, which has a predominance of CD4+ Th17 cells. Therefore, T cell recruitment induced by cuprizone aligns closely with the progression of human MS.^57,61,67^ T cell densities increase in chronic demyelination induced by cuprizone, whereas acute demyelination activates pro-inflammatory pathways leading to oligodendrocyte injury rather than immune cell recruitment. Studies on LPC-induced demyelination support this idea.^57^ As a result, Niraparib modulated T lymphocyte levels to promote the remyelination process.

CD11b, known as the macrophage antigen, indicates the presence of monocytes, granulocytes, and macrophages.^77,78^ The level of CD11b+ cells increased toward the end of CPZ treatment and then decreased upon removal.^23^ The macrophage level in the CPZ group signified the onset of chronic inflammation. In mice treated with Niraparib, a consistent decrease in macrophage levels was observed. Niraparib seems to lower the levels of macrophages in the body. The reduction of macrophage levels during drug treatment emphasizes the noteworthy effect the medication has on inflammation. This relationship highlights the drug’s potential role in modulating immune responses. Additionally, the effect of Niraparib on Gr1+ cells was similar to that on CD11b. Regulatory T cells are naturally occurring suppressor cells that can regulate the immune response to antigens and prevent adverse reactions to them. They can both enhance and inhibit the activity of T cells.^79,80^ Niraparib reduced Treg levels over time after cuprizone was withdrawn. In a previous study (2020), it was determined that regulatory T cells increased for 12 weeks and then decreased at the 16th week. Similarly, the Treg level found in mice that did not receive the drug may be the immune response that started with cuprizone treatment and was regulated by these cells. In mice treated with Niraparib after cuprizone was withdrawn from their diets, the observed decrease in immune response over time may result from the neuroinflammation-reducing effect of Niraparib as an IL-17A inhibitor, alongside the reduction in Treg cells due to the diminished immune response attributed to the presence of the IL-17A inhibitor. In summary, Niraparib altered the levels of macrophages, CD3, T cells, cytotoxic T cells, dendritic cells, APCs, B cells, and other cells, indicating the onset of chronic inflammation in cuprizone control mice, while the levels of regulatory and helper T cells indicate a suppression of inflammation. The differences in the cell levels in blood and in the brain should be carefully considered. The impact of Niraparib on inflammation is indicated by variations in these same cell levels.

Previous studies have reported a reduction in inflammatory cytokines, such as TNF-α and IL-1ß, in the corpus callosum of Act-1 deficient, cuprizone-treated mice. Astrocytes actively produce inflammatory cytokines in response to IL-17 stimulation, playing a crucial role in the progression of cuprizone-induced demyelination.^67^ Secukinumab treatment has previously been shown to prevent the activation of cytokines and other cells by inhibiting IL-17 signaling.^8^ The levels of IFN-gamma increased in the hippocampus and cerebral cortex in response to cuprizone treatment. Secukinumab demonstrated a reduction in the IFN-γ levels.^8^ Additionally, the stimulation of astrocytes by IL-17 produces the cytokines IL-6, TNF alpha, and other chemokines, which recruit leukocytes into the brain.^8,81^ However, Secukinumab treatment recovered the increase in the IL-17 level.^8,67,68,61^ In the compelling study by Avşar et al., the levels of IL-17A exhibited an initial surge, capturing attention with their rise before ultimately tapering off. This dynamic shift underscores the intricate and evolving nature of the immune response, revealing fascinating insights into its functionality. The cuprizone treatment led to a remarkable increase in IFN-γ expression, highlighting an intriguing response worth exploring further.^23,82^ In this study, elevated cytokine levels were observed during the demyelination phase, suggesting that the adverse effects of the drug may indicate its response to inflammation. The maximum levels observed at different weeks during the cuprizone diet indicate a response throughout the demyelination period. This difference has been described as part of an expression cascade.^23^ In this intriguing study, we observed exciting changes in TNF-α, IL-1α, IL-1ß, and IL-17A cytokines following cuprizone intoxication and treatment with Niraparib. Examining cytokine levels after Niraparib administration showed changes that differed from the control group, indicating the drug’s effectiveness. Finally, it can be stated that the impact of Niraparib as a neuroprotective drug is similar to Secukinumab, as the drug reverses the levels of cytokines when comparing Niraparib-treated mice with those in a CPZ-induced model.

In conclusion, this study highlights the potential of repurposing the PARP-1 inhibitor Niraparib as an effective anti-inflammatory agent for the treatment of the MS. The findings suggest that Niraparib not only acts as an anticancer drug but also plays a crucial role in modulating immune responses, particularly by reducing IL-17A-specific inflammation. This mechanism may significantly contribute to decreasing demyelination in conditions such as MS. Further research into the therapeutic applications of PARP-1 inhibitors in neuroinflammatory contexts could open new avenues for managing these challenging diseases.

## Methods

### Molecular Simulations

The 3D molecular structure of IL-17A bound to IL-17RA with PDB ID 4HSA^30^ (available at the time of the study) was chosen for docking and MD studies. Three chains from the original structure were retained, chain A and B, which correspond to the IL-17A dimer and chain C which corresponds to its receptor, IL-17RA. Initially, the Protein Preparation Wizard^83^ from the Maestro molecular modeling suite was utilized to adjust bond orders, add hydrogens, create disulfide bonds and missing side chains from the original structure were completed using Prime.^84^ The Epik module was employed to set protonation states at pH 7.^85^ The hydrogen bond network was optimized and p*K*as were predicted with PROPKA. Restrained minimization was performed using OPLS3e.^86,87^

Grid boxes for docking were created based on previously elucidated critical binding regions of IL-17A to its receptor, IL-17RA. These regions were identified from crystal structures solved by Liu et al., and termed as region i, ii, and iii.^30^ These grid boxes were designed using information from critical residues in region I (S64 and R101) and region II (D42, R55, V65, and W67) at the N-termini, as well as region III (Y85 and H86) and region IV (I127, V128, H129, H130, and V131) at the C-termini of the IL-17A/IL-17RA complex^30^ (Figure 1a). The outer grid box screening distance was set to 30.0 Å and the inner box scanning distance was set to 10.0 Å. The rotation of the thiol and hydroxyl groups in the side chains of the amino acid residues contained in these grid boxes was allowed.

The MetaCore/MetaDrug platform was used to predict the anti-inflammatory effect of Niraparib. The MetaCore/MetaDrug platform reports the predicted anti-inflammatory TAVs by binary QSAR-based models that have been normalized between 0 and 1. A TAV value of 0.5 or higher indicates that the compound possesses therapeutic potential for the disease model being studied. The anti-inflammation is described as follows; training set *N* = 598, Sensitivity = 0.98, Specificity = 1.00, Accuracy = 0.99, Matthews correlation coefficient (MCC)=0.97.^88^

Molecular docking studies were conducted to determine the binding modes and interaction energies between the protein and Niraparib complexes for all 4 grid coordinates. Niraparib was initially prepared at neutral pH using the LigPrep module of the Schrödinger modeling suite (Schrödinger Release 2024-4: LigPrep, Schrödinger, LLC, New York, NY, 2024) with the OPLS3e force field^86,87^ and protonation states at pH 7 were determined using the Epik module. Docking was conducted with Glide/SP, a grid-based docking protocol.^89^ The Glide/SP molecular docking algorithm permits partial flexibility of the ligand and target structure. This allows for the exploration of all possible ligand/protein conformations, thus expanding the interaction network.

Molecular dynamics (MD) simulations were conducted using Desmond,^90^ to investigate the structural and dynamic properties of Niraparib bound complexes. Prior to the MD simulations, ligand and protein complexes in the simulation boxes were solved with the TIP3P water model, a salt concentration of 0.15 M, and neutralized charges, maintaining a buffer distance of 10.0 Å. MD simulations were equilibrated with the Nose−Hoover thermostat and Martyna−Tobias−Klein barostat for the NPT ensemble,^91^ ensuring simulations at 310 K and 1 bar pressure. The simulations were integrated using RESPA^92^ for every 2.0 fs. Short-range electrostatic and van der Waals interactions were calculated within 9 Å of the atom and longer-range interactions were subject to particle mesh Ewald and periodic boundary conditions.^93^ Subsequently, three replicates of 100 ns MD simulations were performed, resulting in the collection of 1000 trajectories from each MD simulation.

### IL-17 Reporter Assay

The inhibitor screening assay was conducted on a recombinant cell line expressing IL-17RA, and the half minimum inhibitory (IC_50_) concentrations were determined. The ability of Niraparib to inhibit IL-17A within cells was tested in HEK 293 cells that were stably transfected with the IL-17RA/IL-17RC heterodimeric receptor and the Act1 adaptor molecule (IL-17 reporter HEK 293 cell, HEK-Blue Invivogen, Cat No: hkb-il17). The IL-17 Reporter Cell Assay was designed to detect bioactive human IL-17A, human IL-17E, and human IL-17F, demonstrating the presence of these cytokines through the activation of the NF-kB and AP-1 pathways. Thus, these cells served as ideal candidates for screening anti-IL-17 and anti-IL-17R antibodies. Additionally, these reporter cells respond to mouse IL-17E, with a minimal response to mouse IL-17A and mouse IL-17F cytokines, but do not respond to mouse IL-17C cytokines. The binding of IL-17A, IL-17E, or IL-17F cytokines to the respective receptors on the surface of HEK-Blue IL-17 cells trigger signaling pathways, initiating the activation of NF-kB and AP-1, allowing for the determination of the ligand−receptor interaction. A total of 3 × 10^4^ HEK-Blue IL-17 cells were added to 96-well plates. IL-17A without an inhibitor was used as positive control. Niraparib was tested at six different concentrations ranging from 1 nM to 100 μM. IC_50_ values were calculated by dose−response inhibition curves with four parameter and nonlinear regression analysis on GraphPad Prism 8 software. Since the cells express IL-17RA and IL-17RC, the inhibitor molecules were added to the wells containing the cells after treatment with the IL-17A molecule provided by the manufacturer. The cells were incubated overnight at 37 °C in a 5% CO_2_ atmosphere. Subsequently, 200 μL of cell medium was treated with 200 μL of QUANTI-Blue solution and read at 630 nm using a multimode reader (Varioskan LUX, Thermo Scientific).

### Toxicity Assay

A toxicity assay was conducted to determine if the inhibitory concentration of Niraparib is toxic to HEK293 cells. MTT (3-(4,5-dimethylthiazol-2-yl)-2,5-diphenyltetrazolium bromide) cell viability/proliferation assay was used. Toxic concentrations causing cell death were demonstrated over a period of 72 h on HEK-Blue IL-17 cell lines in the presence of IL-17A. Cells (2000 cells/well) were triplicated and plated in a suitable 96-well format. Following cell attachment, Niraparib that was dissolved in DMSO, was administered in six different concentrations ranging from 1 nM to 100 μM. Every 24 h, 0.5 mg/mL of MTT solution was added to the medium, and the cells were incubated at 37 °C until visible purple formazan crystals appeared. Subsequently, a solvent solution (10% SDS, 0.01 M HCl) was added, and the samples were incubated at room temperature or 37 °C. After incubation, the cells were measured at 570 nm on a multimode reader (Varioskan LUX, Thermo Scientific). Additionally, photographs of the cells were taken prior to the MTT application.

### Cuprizone Model

C57BL/6 were acclimated for a week before experiments began, and toxic demyelination model was induced by feeding mice with 0.4% (w/w) cuprizone (bis-cyclohexanone oxaldihydrazone, Sigma-Aldrich) for 8 weeks. Temperature (25 ± 2 °C) and humidity (50 ± 10%) were maintained at appropriate intervals, during 12 h light−dark cycles. The animals were kept in groups of 6 per cage. The mice were maintained on a rodent diet and with tap water given. Cuprizone (CPZ) was mixed into a ground standard rodent chow. CPZ diet was maintained for 8 weeks until complete chronic demyelination, then mouse was fed with normal rodent chow. Experimental cages set up as two groups; (i) CPZ control group was fed with CPZ added chow for 8 weeks, then fed with normal chow until 16th week; (ii) Niraparib group was fed with fed with CPZ added chow for 8 weeks, then fed with normal chow until 16th week after Niraparib treatment. Experimental and control mice were analyzed every 2 weeks. For analysis of remyelination, mice were fed with normal chow for 8 weeks following 8 weeks of CPZ administration. Animals were deeply anesthetized and perfused with ice-cold phosphate buffered saline (PBS) and 4% paraformaldehyde (PFA). For the mouse groups receiving the drug, a total of 2 mg/mL of Niraparib was administered via gavage, dissolved in 1× PBS, immediately following an 8-week cuprizone treatment for a duration of 5 days. The CPZ control group mice received a PBS solution under the same volume and conditions.

### Immunohistochemistry Staining for Demyelination

Immunohistochemistry (IHC) was utilized to monitor the demyelination lesions targeted in the brain following an eight-week cuprizone diet, as well as the expected remyelination resulting from subsequent feeding with normal mouse chow. The myelination process in the corpus callosum and cerebellum regions of the mouse brains was examined after the animals were sacrificed and their brains extracted by performing perfusion brains were embedded in paraffin blocks. Paraffin-embedded tissues were sectioned at 3 μm thickness. Sections of these brains were stained with Hematoxylin and Eosin (H&E), Luxol Fast Blue (LFB) for routine histology. Sections were incubated at 65 °C for 1 h for deparaffinization. After being cleaned with xylene four times for 5 min each, followed by ethanol (EtOH) four times for 5 min, the sections were washed with water and prepared for antigen retrieval. The sections were placed in a plastic dish and incubated with the 1× EnVision FLEX Target retrieval Solution, High pH (50×) (Dako Omnis). After being microwaved, the sections were allowed to cool to room temperature and then washed with distilled water (dH_2_O). Peroxidase blocking was performed using 3% hydrogen peroxide, and the sections were then washed again with distilled water. Primary antibodies were applied to the sections and incubated in the dark at room temperature for 1−2 h. 1:200 Myelin Basic Protein (MBP) (Cat No: E-AB-70265, Elabscience), 1:250 GFAP (sc-33673, Santa Cruz Biot.), 1:500 IL-17A (Cat No: PA5-79470, Thermo Fisher), and mouse monoclonal 1:500 RBFox3/NeuN (Cat No: NBP1-92693, Novus) were used as primer antibodies. Following washing with PBS-T for 3−5 min, HRP-conjugated secondary antibodies (Cat No: 7074S and 7076S, CST) were applied and incubated in the dark at room temperature for another 1−2 h. After incubation, the sections were washed with PBS-T and treated with EnVision FLEX DAB solution (Dako Omnis, DAB+Substrate Chromogen solution) for 10 min at room temperature. The sections were then washed with PBS-T and incubated in hematoxylin for 30 s before being rinsed with water. After washing with PBS-T and water, the sections were treated with ethanol (EtOH) and then allowed to dry before being mounted with a coverslip. The brain sections were photographed under a microscope. Morphology of the tissues were monitored at 10× under light microscope to demyelination.

### Immunological Marker Analysis with Flow Cytometry

Blood samples collected from the tails of mice were prepared for flow cytometry measurements on the same day using PBS/EDTA tubes. The blood collected in PBS/EDTA solution was centrifuged at 1450 rpm for 5 min and pellet was lysed by using 400−500 μL red blood cell (RBC) lysis buffer (155 mM NH_4_Cl, 10 mM KHCO_3_ and 127 mM EDTA). A 1× HBSS solution containing 2% FBS was then added to fill the tubes, followed by centrifugation. Antibodies were added to the pellet, pipetted gently, and incubated in the dark at +4 °C for 20 min. After incubation, an additional 1x HBSS solution with 2% FBS was added to fill the tubes and centrifuged again. The supernatant was discarded, and the pellet was homogenized with 200 μL of 1× HBSS containing 2% FBS for flow cytometry measurement. For cells stained with Foxp3 antibody, after the RBC solution treatment, a permeabilization buffer was added to the cells and incubated at room temperature for 15 min. Subsequently, a neutralization buffer was added, followed by a 15 min incubation at room temperature and centrifugation at 500*g* for 5 min. After washing the cells with 1× HBSS containing 2% FBS, the antibodies were prepared at the designated dilution and added to the cells, followed by incubation in the dark at +4 °C for 20 min. After incubation, the cells were washed again and homogenized with 200 μL of 1× HBSS containing 2% FBS for flow cytometry measurement.

Marker used to determine inflammation with optimized dilutions: APC antimouse CD4+ (Cat No: 100515, Biolegend), Alexa Fluor700 antimouse CD8a+ (Cat No: 100729, Biolegend), FITC antimouse CD8b+ (Cat No: 126605, Biolegend), PE antimouse CD3+ (Cat No: 100205, Biolegend), Alexa Fluor700 antimouse Foxp3 (Cat No: 126421, Biolegend), PE/Cy5 antimouse/human CD45R/B220 (Cat No: 103209, Biolegend), APC antimouse CD48 (Cat No: 103411), FITC antimouse Ly-6G/Ly6C (Gr-1) (Cat No: 108405, BioLegend), PE antimouse CD86 (Cat No: 159203, Biolegend), APC/Cy7 antimouse/human CD11b (Cat No: 101225, Biolegend) and PE antimouse CD25 (Cat No: 101903, Biolegend).

### LEGENDplex Mouse Inflammation Panel

The LEGENDplex Mouse Inflammation Panel (BioLegend, Cat No: 740446) is a multiplex assay performed using fluorescence. It contains suitable beads designed for measurement in flow cytometry. This cytokine panel assesses 13 mouse cytokines: IL-1α, IL-1β, IL-6, IL-10, IL-12p70, IL-17A, IL-23, IL-27, MCP-1, IFN-β, IFN-γ, TNF-α, and GM-CSF. All solutions provided with the kit were brought to room temperature and prepared according to the manufacturer’s specified conditions. To begin, 25 μL of Assay Buffer and 25 μL of plasma samples were added to each well, while 25 μL of standards was combined with 25 μL of Matrix C solution in the wells. Concurrently, the cytokine beads provided by the kit were vortexed for 30 s and then 25 μL of these beads were added to each well, achieving a total volume of 75 μL. The wells were covered and incubated in the dark at room temperature, shaking at 800 rpm for 2 h. Following this, the samples were centrifuged at 1050 rpm for 5 min, and the supernatant was discarded immediately. After washing the wells with 200 μL of Washing Buffer, they were centrifuged again at 800 rpm for 1 min, and this washing step is repeated. Next, 25 μL of Detection Antibodies were added to each well. The wells were covered again and incubated in the dark at the same shaking conditions for 1 h. Without washing, 25 μL of SA-PE solution was added to each well, and the wells were once more covered and shaken under the same conditions for 30 min. After repeating the washing step, 150 μL of 1× Wash Buffer was added to each well, and the beads were mixed by pipetting before reading them on the flow cytometry device. The flow setup for measuring the samples was conducted as specified in the kit instructions.

### Statistical Analysis

Statistical analysis was performed using the *scipy* and *scikit-posthocs* libraries in Python 3.8. For comparison of cell surface markers and cytokine panels as well as cytokine immunohistology staining and percentages of myelinated axons on the microscope, statistical comparison of more than two histological score groups was performed with the Kruskal−Wallis test followed by Dunn’s posthoc. We used the Mann−Whitney test to compare the medians of two independent groups. In all cases, *p* < 0.1 was considered as statistically significant. † indicated *p* < 0.1, * indicated *p* < 0.05, ** indicated *p* < 0.01, *** indicated *p* < 0.001.

## Supplementary Material



## Data Availability

No additional data is available. All data and additional data provided as Supporting Information. The raw data supporting the conclusion of this article will be made available by the authors upon reasonable request. No data sets were generated or analyzed during the study.

## Notes

The colorimetric inhibitory effects of Niraparib on HEK-Blue IL-17 cells were investigated as depicted in Figure S1. Additionally, morphological images were captured to assess the cell viability of HEK cell lines following a 24 h treatment with Niraparib, as shown in Figure S2. The study also included a flow cytometric analysis of regulatory T cells (Tregs) in the cuprizone (CPZ) model. This analysis utilized both the CD25+/Foxp3+ double marker and the CD4+/CD25+/Foxp3+ triple marker to evaluate the impact of Niraparib treatment, with the results detailed in Figure S3 (PDF)

## Notes

Ethics Approval: Animal experiments were conducted in accordance with the guidelines of the animal unit committee at Marmara University Experimental Animal Studies Facility Ethical Board (DEHAMER, Protocol No: 11.2023mar). All experiment procedures were carried out by the guidelines of the Ethics Committee of the Bahçeşehir University, Turkey.
