# Supplementary material for: Niraparib Demonstrates Therapeutic Potential in Multiple Sclerosis through Inhibition of IL-17A Receptor Interaction and Promotion of Remyelination
Source: ACS Chem Neurosci. 2025 Sep 9;16(19):3816–33. doi: 10.1021/acschemneuro.5c00519 (PMC12498388; doi:10.1021/acschemneuro.5c00519)
Supplement: Supplementary file 1 [file cn5c00519_si_001.pdf]

Supporting Information

Niraparib Demonstrates Therapeutic Potential in Multiple Sclerosis Through Inhibition of IL17A Receptor Interaction and Promotion of Remyelination

Muge Didem Orhan[1,2,3], Lalehan Oktay[4,5], Ayşe Irem Cınar[6], Aybek Kagan Yesil[6], Huseyin Tunc[7], Fatih Eren [8,9,10], Serdar Durdagi[4,5,11], Timucin Avsar[*2,3]

[1] Neuroscience, Graduate School of Education, Bahçeşehir University, Istanbul, Turkey

[2] Department of Medical Biology, Faculty of Medicine, Bahçeşehir University, Istanbul, Turkey

[3] Neurooncology Laboratory, Faculty of Medicine, Bahçeşehir University, Istanbul, Turkey

[4] Computational Biology and Molecular Simulations Laboratory, Department of Biophysics, Faculty of Medicine, Bahçeşehir University, Istanbul, Turkey

[5] Lab for Innovative Drugs (Lab4IND), Computational Drug Design Center (HITMER), Bahçeşehir University, Istanbul, Turkey

[6] Neuroscience Program, University of Bonn, Bonn, Germany

[7] Department of Biostatistics and Medical Informatics, Bahçeşehir University, Istanbul, Turkey

[8] Department of Medical Biology, School of Medicine, Marmara University, Istanbul, Türkiye

[9] Faculty of Medicine, Recep Tayyip Erdoğan University, Rize, Türkiye

[10] Institute of Gastroenterology Liver Research Unit, Marmara University, Türkiye

[11] Molecular Therapy Lab, Department of Pharmaceutical Chemistry, School of Pharmacy, Bahçeşehir University, Istanbul, Turkey

Corresponding author e-mail: timucin.avsar@bau.edu.tr

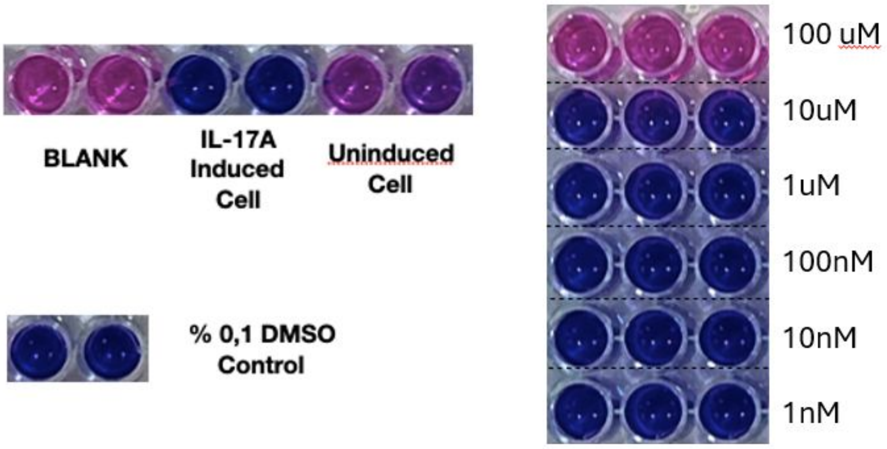

Figure S1: Inhibitory effects of Niraparib on HEK-Blue IL-17 cells. The inhibition of IL-17A/IL-17RA interaction by Niraparib in HEK-Blue IL-17 cells was evaluated using the reporter assay. The intensity of the purple-blue color in the wells indicates the level of SEAP (secreted alkaline phosphatase) activated by IL-17A/IL-17RA binding. The "IL-17A induced cell" well represents positive control without any inhibitor, showing maximum SEAP activation (blue color). The "Uninduced cell" well represents the negative control without IL-17A, showing minimal SEAP activation (light purple color). The inhibitory effects of Niraparib at 100 µM (high concentration) and 1 µM (low concentration) are determined by the decrease in color intensity. The reduction in color intensity demonstrates that Niraparib inhibits IL-17A/IL-17RA interaction in a dose-dependent manner.

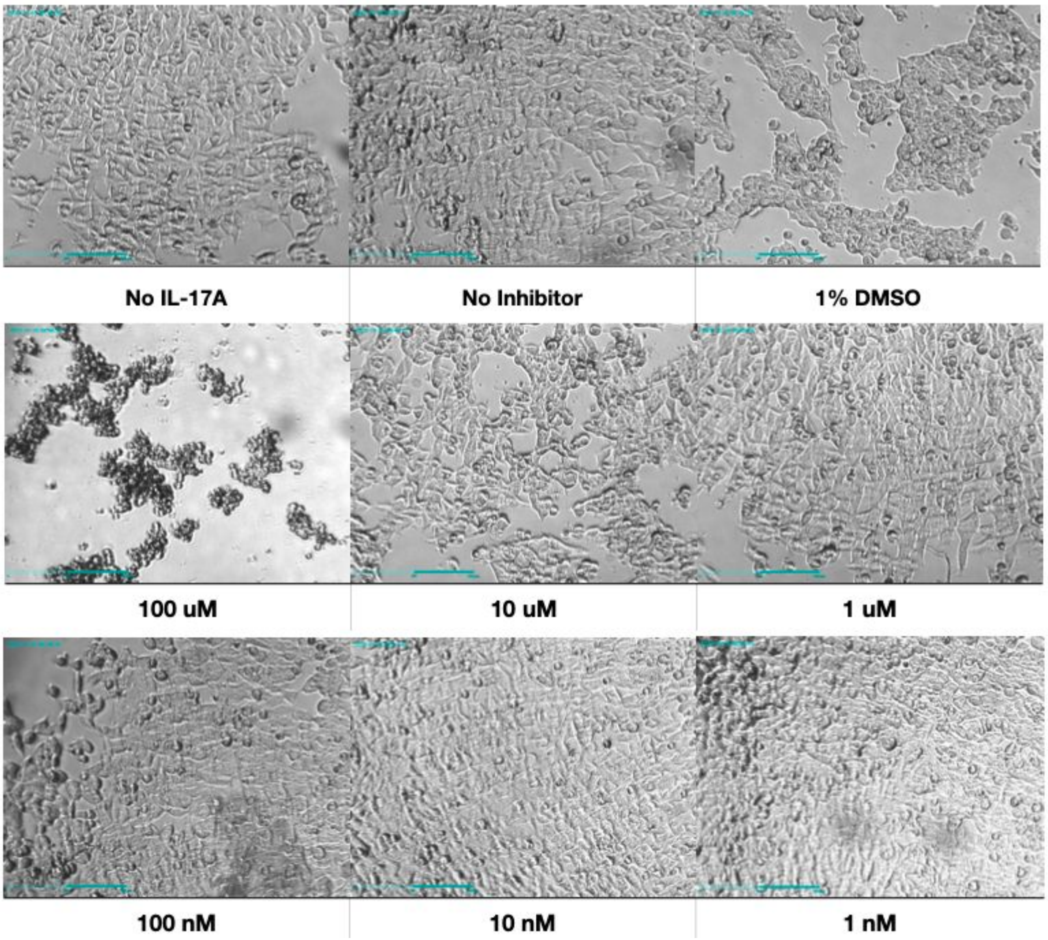

Figure S2: Cell viability of HEK cell lines treated with Niraparib for 24 hours. This figure presents the cell viability of HEK cell lines following 24 hours of treatment with varying concentrations of Niraparib. The first three images depict control conditions: "No IL-17A" represents cells without IL-17A induction, serving as a baseline; "No Inhibitor" represents cells induced with IL-17A but without Niraparib treatment, indicating the effect of IL-17A alone; and "%1DMSO" represents cells induced with IL-17A and treated with 1% DMSO, serving as vehicle control. Subsequent images show the cell viability at different Niraparib concentrations, allowing for the assessment of its impact on HEK cell viability in the presence of IL-17A.

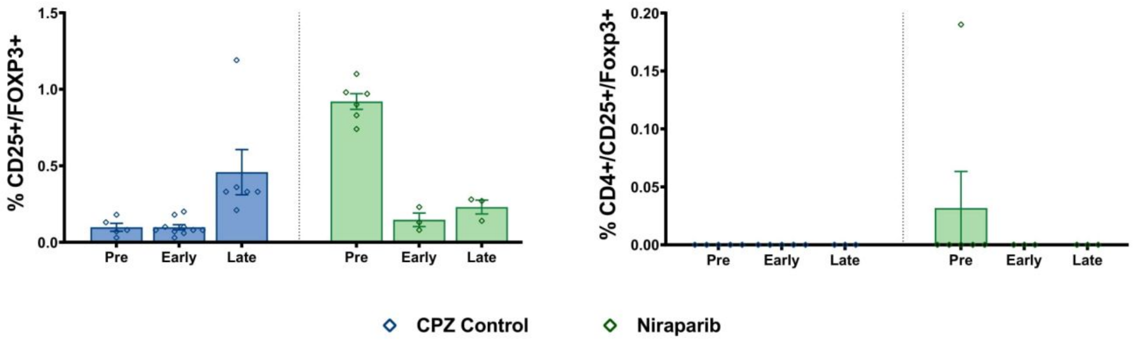

Figure S3: Flow cytometric analysis of regulatory T cells (Tregs) in the cuprizone (CPZ) model with and without Niraparib treatment. Data was derived from blood. Blue and green bars indicate cuprizone-treated (untreated) and Niraparib treated mice groups respectively. X- axis represents the weeks that include receiving treatment (Early: 0-8 weeks and Late 8-16 weeks). Differences were determined using Kruskal-Wallis Test followed by Mann-Whitney test to compare the mediand of two independent groups. The following symbols are used to indicate the level of significance: † for $p<0.1$, * for $p<0.05$, ** for $p<0.01$, *** for $p<0.001$.
